# Different Oxidative Stress and Inflammation Patterns of Diseased Left Anterior Descending Coronary Artery versus Internal Thoracic Artery

**DOI:** 10.3390/antiox13101180

**Published:** 2024-09-28

**Authors:** Andrea Salica, Vittoria Cammisotto, Raffaele Scaffa, Giulio Folino, Ruggero De Paulis, Roberto Carnevale, Umberto Benedetto, Wael Saade, Antonino Marullo, Sebastiano Sciarretta, Gianmarco Sarto, Silvia Palmerio, Valentina Valenti, Mariangela Peruzzi, Fabio Miraldi, Francesco Giosuè Irace, Giacomo Frati

**Affiliations:** 1 Department of Cardiac Surgery, European Hospital, 00149 Rome, Italy; 2 Department of Clinical, Internal Anesthesiological and Cardiovascular Sciences, Sapienza University of Rome, Viale del Policlinico, 155, 00161 Rome, Italy; 3 UniCamillus, International University of Health Sciences, Rome, Italy; 4 Department of Medico-Surgical Sciences and Biotechnologies, Sapienza University of Rome, Corso della Repubblica 79, 04100 Latina, Italy; 5 IRCCS NeuroMed, 86077 Pozzilli, Italy; 6 Department of Cardiac Surgery, University “G. d’Annunzio”, 66013 Pescara, Italy; 7 Maria Cecilia Hospital, GVM Care & Research, 48010 Cotignola, Italy; 8 Department of Cardiac Surgery and Heart Transplantation, San Camillo Forlanini Hospital, Circ.ne Gianicolense 87, 00152 Rome, Italy

**Keywords:** internal thoracic artery, left anterior descending artery, coronary artery bypass graft, oxidative stress, inflammation

## Abstract

Background. Oxidative stress and inflammation are typically implied in atherosclerosis pathogenesis and progression, especially in coronary artery disease (CAD). Our objective was to investigate the oxidative stress and inflammation burden directly associated with atherosclerotic plaque in patients with stable coronary disease undergoing coronary artery bypass graft (CABG) surgery. Specifically, markers of oxidative stress and inflammation were compared in blood samples obtained from the atherosclerotic left anterior descending artery (LAD) and blood samples obtained from the healthy left internal thoracic artery (LITA), used as a bypass graft, within the same patient. Methods. Twenty patients scheduled for off-pump CABG were enrolled. Blood samples were collected from the LITA below anastomosis and the LAD below the stenosis. Samples were analysed for oxidative stress (sNOXdp, H_2_O_2_, NO) and inflammation markers (TNFα, IL-6, IL-1β, IL-10). Results. The analysis showed a significant increase in oxidative stress burden in the LAD as compared to LITA, as indicated by higher sNOX2-dp and H_2_O_2_ levels and lower NO levels (*p* < 0.01). Also, pro-inflammatory cytokines were increased in the LAD as compared to the LITA, as indicated by higher TNFα and IL-6 amounts (*p* < 0.01). On the other hand, no significant differences could be seen regarding IL-1β and IL-10 levels between the two groups. Conclusions. The oxidative stress and inflammatory burden are specifically enhanced in the LAD artery of stable coronary patients compared to systemic blood from the LITA of stable coronary patients.

## 1. Introduction

Cardiovascular (CV) diseases represent the leading cause of morbidity and mortality worldwide due to coronary artery disease (CAD) and subsequent heart failure, and atherosclerosis is the most common underlying pathology of CAD, peripheral artery disease, and cerebrovascular disease [1,2,3,4]. Despite efforts in terms of lifestyle modifications, primary prevention, and conventional as well as innovative pharmacological therapies, including lipid-lowering and anti-hypertensive drugs, the incidence of clinical events derived from atherosclerotic disease remains high [4,5]. Systemic and vascular inflammation play a crucial role in the genesis of atherosclerosis, the occurrence of cardiovascular events and the severity of clinical outcomes [6,7,8,9,10], with the therapeutic role of anti-inflammatory interventions recently demonstrated in large clinical trials with drugs targeting specific inflammatory mechanisms [11,12,13]. Therefore, additional efforts are needed to understand the inflammatory and oxidative mechanisms underlying atherosclerosis development. Human studies linking specific markers of inflammation and oxidative stress with coronary atherosclerosis, by directly measuring these markers in diseased and healthy vessels, would significantly help to better understand these pathophysiological processes. Thus, we analyzed the concentration of pro-inflammatory molecules and oxidative stress markers in blood obtained from the left ITA (LITA) and native coronary artery—left anterior descending artery (LAD)—in patients undergoing off-pump coronary artery bypass graft (OPCABG). Of note, LITA is not only resilient to atherosclerosis representing a benchmark conduit for this study [14,15,16].

## 2. Materials and Methods

### 2.1. Study Design

This study was conducted at the European Hospital of Rome from June 2021 to July 2022. Patients scheduled for surgical coronary artery revascularization were enrolled. Inclusion criteria were an age between 18 and 85 years old, an off-pump procedure, the utilization of in situ LITA, and informed consent. Patients with recent STEMI (<30 days), severe left ventricular dysfunction (LVEF < 30%), acute or chronic inflammatory diseases, rheumatological or immunological diseases, active malignancy, or active infections were excluded. To ascertain the required sample size, we utilized G*Power v3.1 software, with the assumption of a mean difference of approximately 50% in major outcome variables between the two groups (LITA vs. LAD).

The choice to include only off-pump procedures was aimed at avoiding the impact of a cardiopulmonary bypass (CPB) on inflammation and oxidative stress homeostasis. For each patient, we collected demographic data, and clinical and anamnestic information, and also performed routine blood tests. All patients received preoperatory coronary angiography, transthoracic echocardiography, chest X-rays, and ultrasounds of supra-aortic vessels.

This study was registered at ClinicalTrial.gov: https://clinicaltrials.gov/study/NCT05574621?term=NCT05574621&rank=1 (accessed on 23 September 2024) (NCT05574621) and was approved by the European Hospital Ethical Committee (N° 2021-01).

### 2.2. Surgical Procedure

All patients enrolled underwent surgical coronary artery revascularization. The procedure was performed under general anaesthesia, via full median sternotomy. The left internal thoracic artery was harvested in skeletonized fashion; the saphenous vein was harvested with open standard technique; full-dose heparinization was administered (300 UI/kg); and the heart was stabilized with an Octopus (Medtronic, Minneapolis, MN, USA) device. Distal anastomoses were performed starting from LITA-LAD; subsequently, other bypass grafts were performed using the right internal thoracic artery (RITA) and/or the saphenous vein graft (SVG); and proximal anastomosis of free grafts was performed on the ascending aorta with partial clamping. Heparin was antagonized with protamine sulphate; haemostasis was obtained; and the chest was closed in a standard fashion.

### 2.3. Blood Sampling

After heparinization, blood samples (10 mL) were collected directly from the distal edge of LITA: after completing the artery harvesting, the distal edge was ligated and cut, and the blood was directly spilled into a sterile container and collected. Immediately after, LAD arteriotomy was performed and blood dripping/jet was collected directly with a syringe; blood from LITA and LAD was then transferred into respective vacutainer tubes without an anticoagulant to obtain serum samples. Serum samples, obtained by centrifuging the blood at 300× *g* for 10 min, were stored at −80 °C until the time of the analysis.

### 2.4. Oxidative Stress Assays

Serum sNOX2-dp. NOX2 activation was measured in serum samples as soluble NOX2-derived peptide (sNOX2-dp) with an ELISA method, as previously described [17]. Briefly, the peptide was recognized by binding to a specific monoclonal antibody against the amino acid sequence (224–268) of the external portion of NOX2, which was released following platelet activation. Values were expressed as pg/mL; intra-assay and inter-assay coefficients of variation were 8.95% and 9.01%, respectively.

Serum H_2_O_2_ evaluation. Hydrogen peroxide (H_2_O_2_) in serum samples was assessed by a Colorimetric Detection Kit (Arbor Assays, Ann Arbor, MI, USA). Values were expressed as μM. Intra-assay and inter-assay coefficients of variation were 2.1% and 3.7%, respectively.

Serum nitric oxide bioavailability. Nitric oxide (NO) bioavailability in serum samples was determined by a colorimetric assay kit (Cell Biolabs, San Diego, CA, USA) that quantitatively measured NO by NO_2_^−^/NO_3_ calculation. Total nitrite was detected with Griess Reagents as a coloured dye product (absorbance 540 nm). The concentration was expressed as μM. Intra- and inter-assay coefficients of variation were <10%.

### 2.5. Cytokines Determination

TNF-α assay. TNFα blood levels were evaluated in serum samples by a commercial immunoassay kit (Abcam), and values were expressed as pg/mL; intra- and inter-assay CVs were <10%.

IL-1β assay. The concentration of interleukin 1β (IL-1 β) in serum samples was measured using an Enzyme-Linked Immunosorbent Assay kit (Abcam, Cambridge, UK). The values were expressed in pg/mL Intra- and inter-assay CV were <12% and <10%, respectively.

IL-6 assay. An IL-6 Enzyme-Linked Immunosorbent Assay kit was used to determine the quantitative measurement of IL-6 protein in serum samples. Values were expressed as pg/mL. Intra-assay and inter-assay coefficients of variation were 2.1% and 2.4%, respectively.

IL-10 assay. The serum determination of IL-10 was performed by an Enzyme-Linked Immunosorbent Assay kit (Abcam). Values were expressed as pg/mL. Intra-assay and inter-assay coefficients of variation were 3.2% and 7.3%, respectively.

### 2.6. Statistical Analysis

To ascertain the required sample size, we utilized G*Power v3.1 software, with the assumption of a mean difference of approximately 50% in major outcome variables between the two groups (LITA vs. LAD). This assumption was derived from a pilot comparison analysis on IL-6 only.

All data processing was executed on a workstation running IBM-SPSS 26 (Armonk, NY, USA) on a Windows 10 machine (Microsoft Corp, Redmond, WA, USA). Categorical variables were presented as numbers and percentages and were analysed by Pearson’s Chi-squared or Fisher’s exact test. Continuous variables were expressed as mean with standard deviation (SD) or median with interquartile range (IQR). Normality of the data were assessed using the Shapiro–Wilk test. Differences between groups were compared using the paired Student’s *t*-test. Correlation analysis and subsequent univariate/multivariate regression was run to identify possible confounders (Supplementary Material S1).

## 3. Results

### 3.1. Patients

Twenty patients were enrolled in the study, and the baseline characteristics are summarized in Table 1. The mean age was 65 ± 10 years old; 18 (90%) were males. All told, 8 patients had diabetes (40%), 16 were former or active smokers (80%), 17 had hypertension (85%), and 15 had dyslipidaemia (75%). Coronary angiography showed a multivessel coronary pathology in all patients, with a median of 4 ± 1 vessels involved and a median Syntax score of 24 ± 8; all patients had stenosis in the LAD (median 80%, range 70–100%). Preoperative echocardiography showed a median LVEF of 60% ± 7%. (Preoperative echocardiography and coronary angiography findings are summarized in Table 2). Preoperative blood samples showed a median of 8200 ± 2600/µL WBC, a CRP of 0.16 ± 0.38 mg/dL, and an ESR of 11 ± 14 s. (Comprehensive preoperative blood sample results are summarized in Supplementary Table S1). Overall, 17 patients (85%) were on acetylsalicylic acid (ASA), 3 (15%) on clopidogrel (CPD), 17 (85%) on statins and 17 (85%) on ACE-inhibitors or angiotensin II receptor blockers at admission (Supplementary Table S2).

### 3.2. Procedure Outcome

All patients underwent off-pump coronary artery surgical revascularization as scheduled, and all received an in situ LITA-LAD graft. Patients received a 3 ± 1 graft, and double thoracic artery was used in 9 (45%) patients. One procedure was later converted (on-pump) to re-perform the LITA-LAD anastomosis. There were no intraoperative or perioperative deaths. Four patients (20%) suffered post-operative transient atrial fibrillation.

### 3.3. Laboratory Findings

#### 3.3.1. Oxidative Stress

**Figure 1:**
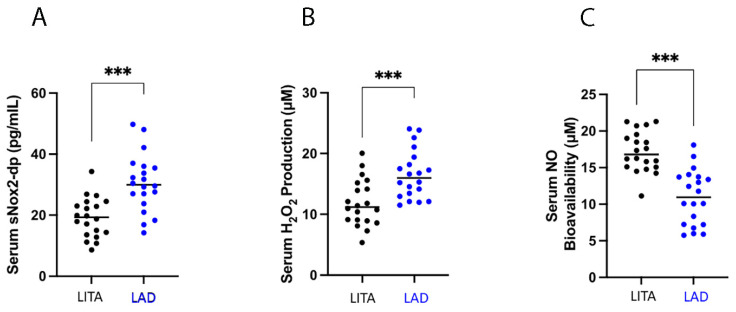
Diagram showing different distribution between (left internal thoracic artery) LITA and (left anterior descending) LAD blood samples: (**A**): sNOX2-dp; (**B**): H_2_O_2_; (**C**): NO. Data are expressed as mean and standard deviation (SD); *** p < 0.0001.

Blood sampled from the LITA showed lower levels of oxidative stress parameters compared to blood sampled from the LAD. In particular, the LITA group showed lower levels of sNOX2-dp compared to the LAD group (19.22 ± 6.49 vs. 30.57 ± 9.46 pg/mL, *p* < 0.001, respectively) (Figure 1A).

Accordingly, the LITA group had lower levels of H_2_O_2_ production than the LAD group (11.88 ± 3.82 vs. 16.54 ± 3.98 µM, *p* < 0.001, respectively) (Figure 1B).

In addition, as increased production of ROS leads to oxidative stress and NO reduction [18], NO availability was also evaluated. Specifically, the LITA group showed a better endothelial status, as demonstrated by the significantly higher levels of NO availability in the LITA group compared to the LAD group (17.18 ± 2.76 vs. 10.95 ± 3.75 µM, *p* < 0.001, respectively) (Figure 1C).

#### 3.3.2. Inflammatory Status

**Figure 2:**
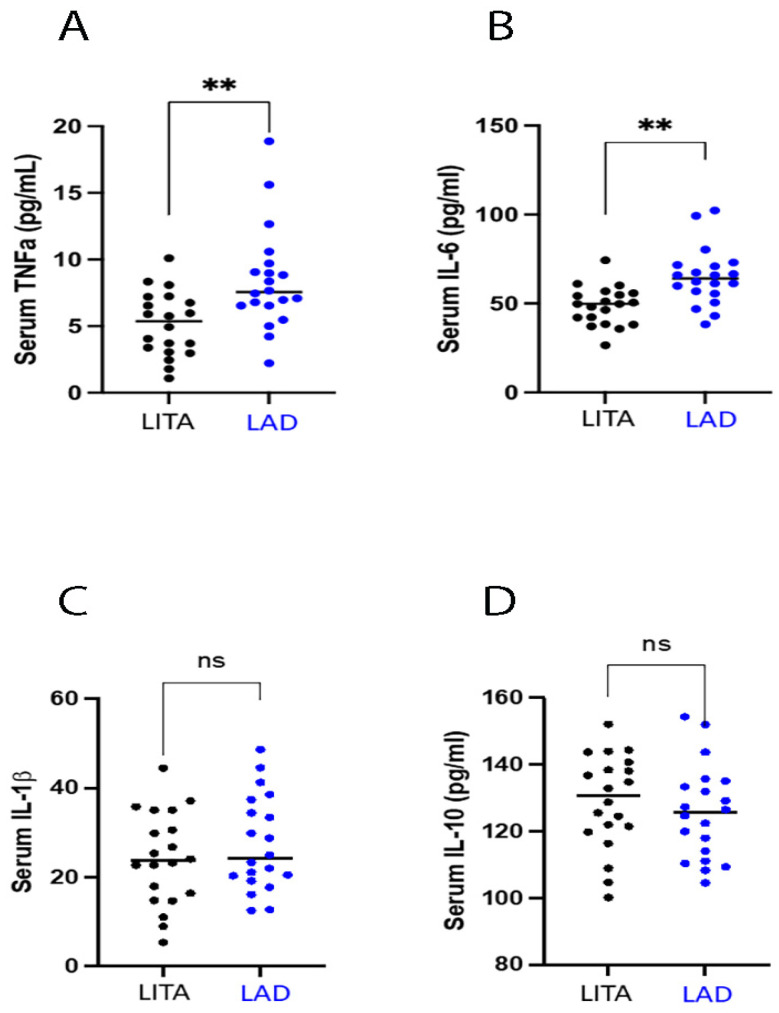
The diagram shows different distribution between (left internal thoracic artery) LITA and left anterior descending) LAD blood samples: (**A**): TNFα; (**B**):IL-6; (**C**):IL-1β; (**D**): IL-10. Data are expressed as mean and standard deviation (SD); ** p < 0.001; ns = not significant.

For the evaluation of inflammatory status, we measured the pro- and anti-inflammatory cytokines. Specifically, in LITA group, we found reduced levels of TNF-α and IL-6 compared to the LAD group (5.15 ± 2.42 vs. 8.43 ± 3.82 pg/mL, *p* < 0.05, for TNF-α and 48.71 ± 10.84 vs. 65.00 ± 16.06 pg/mL, *p* < 0.05, for IL-6) (Figure 2A,B). Conversely, no changes were observed between the two groups both in IL-1β levels (24.05 ± 10.45 vs. 27.33 ± 10.70 pg/mL, *p* = 0.109) and the anti-inflammatory cytokine IL-10 (128.84 ± 14.21 vs. 125.54 ± 14.23 pg/mL, *p* = 0.353) (Figure 2C,D).

## 4. Discussion

Oxidative stress occurs when the excessive production of reactive oxygen species (ROS) sweeps endogenous antioxidant defenses, resulting in tissue injury [19]. Studies based on analyses of human atrial tissues collected during cardiac surgery suggest a pathogenic role of oxidative stress and identified elevated atrial nicotinamide adenine dinucleotide phosphate (NADPH) oxidase activity associated with post-operative complications [20,21]. There is also evidence of the role of pathogenic oxidative stress in atherotrombotic coronary artery disease [18].

In this context, data measuring circulating biomarkers of oxidative stress have yielded mixed results [22,23] and there is a lack of comprehensive evaluation of the levels of oxidative stress in local districts, particularly in the coronary arteries. In this study, we demonstrated that LAD-derived blood shows a deranged inflammatory and oxidative marker profile as compared to systemic blood from the LITA, with increased NOX2-mediated oxidative stress, reduced NO availability, and a potentially augmented inflammatory burden. In particular, our data showed that in blood derived from the LAD the NOX-2, the main source of ROS [24], plays a pivotal role as a mediator of oxidative stress and seems to be involved in enhanced ROS production in this district, as highlighted by an increase in sNOX2-dp and H_2_O_2_ levels observed in LAD samples.

Concurrently, we detected a reduction of NO levels in LAD samples as compared to blood sampled from LITA. This result confirms a worse endothelial status in LAD. In fact, NO is one of the primary mediators of endothelium-dependent vasodilatation [25], and in the vessel walls NO may be scavenged by an elevated level of ROS, produced primarily by NADPH oxidase [26].

Of note, the differences observed are statistically significant, even though we are dealing with a small sample of patients.

Endothelial dysfunction is strongly associated with oxidative stress, as well as with vascular inflammation, representing a unified mechanism for the underlying pathophysiology of cardiovascular morbidity and mortality [27]. In fact, several pro-inflammatory cytokines, including interleukin (IL)-1β, IL-6, and tumor necrosis factor-alpha (TNFα), as well as anti-inflammatory cytokines, such as IL-10, have been identified as part of the inflammatory process of artery walls [28,29]. Serum levels may be elevated in patients with CAD and have been found to predict cardiovascular risk [30,31].

The alteration of systemic inflammatory burden in patients with coronary artery disease has been proven in numerous studies. Twenty years ago, Uzui and colleagues [32] showed the augmented inflammatory markers levels inside the coronary atherosclerotic plaques, especially those of metalloproteinases, oxidated lipids, and TNFα. The study, even if it is a milestone, was performed post-mortem on autoptic samples. The FINRISK study [33] showed further association between cardiovascular risk and systemic inflammation, as evaluated by CRP and TNFα. More recently, Jong-Hwa Ahn and colleagues [9] published a comprehensive study on the residual cardiovascular risk after a first cardiovascular event, again associated with systemic inflammation. Finally, the CANTOS trial group confirmed the interaction of CRP, interleukin-1 β, interleukin-6 and interleukin-18, and cardiovascular events [11,12,34]. We believe that our study significantly extends this work: with samples collected directly from the LAD (without interference from different districts), we were able to demonstrate that NOX2-derived oxidative stress may directly contribute to inflammation, endothelial dysfunction, and coronary atherosclerotic disease. This is evidenced by the fact that the use of natural products such as trehalose, spermidine, catechin, epicatechin, and oleuropein can inhibit NOX-2-mediated oxidative stress and improve endothelial function, thereby reducing the impact of risk factors on the development of cardiovascular disease [35,36]. A direct analysis of the differences regarding inflammatory and oxidative stress in diseased coronary arteries and healthy internal thoracic arteries allowed us to consistently hypothesize a direct association between atheromatic plaque and biological response.

Previous studies focused on the comparison between coronary and systemic blood with samples drawn from the coronary sinus during on-pump bypass surgery or during hemodynamic procedures through coronary catheters, but always located either in the coronary sinus or upstream of the atherosclerotic stenosis/plaque [37,38].

Another recent study, more similar to ours, compared the metabolome of aortic blood and internal thoracic artery blood. However, the analysis performed was an “untargeted metabolomic”, which therefore found no particular molecules except an increase in methionine and cysteine in the LITA group [39].

In this study, we found significantly higher levels of TNFα and IL-6 in LAD blood when compared to LITA samples.

On the other hand, IL-1β and IL-10 did not show any difference. Of note, IL-10 is considered an anti-inflammatory cytokine, rising in the late inflammatory phase and facilitating inflammation resolution, tissue clearance, and healing [40]. We may speculate that TNFα and IL-6 may play a primary role in the promotion of coronary atherosclerosis, although the lack of a time-course observation in our experimental setting did not allow us to support this assumption with data [41,42].

However, it should be noted that the increase in Il-6 represents a link between inflammation process and coronary atherosclerosis as has been attempted by several clinical trials. In particular, IL-6 inhibition safely and effectively reduces biomarkers of inflammation and thrombosis in patients at high cardiovascular risk. Indeed, based on the recent RESCUE results [41], the opportunity has arisen to move beyond CANTOS [11] and IL-1β inhibition and to investigate in ZEUS [43] whether targeting IL-6 can provide an even greater reduction in cardiovascular event rates acting on residual inflammatory risk.

### 4.1. Possible Translational Implications in Coronary Surgery

Since revascularization with an in situ LITA carries a significantly lower risk of not only conduit disease progression, but also downstream coronary disease progression (over stents and other conduits), improved patient outcomes with LITA grafting could be attributed to a combination of better graft patency itself, as well as decreased atherosclerosis progression downstream of the anastomosis performed [14,15]. As evidence of this, several reports have shown coronary plaque regression following a LITA graft to the LAD, with non-infrequent complete resolution of visible plaque [16,44,45].

We speculate that the benefits of surgical coronary revascularization go beyond simple emorheology, but also offer a biological advantage in preventing hte progression of atherosclerosis through a positive modulation of oxidative stress and inflammation pathways. 

Upon the activation of the bypass, not only adequate hemodynamic will be reestablished downstream of the stenosis, but also healthy blood flow will be restored with the consequence that all described biological changes may have positive effects in terms of oxidative stress and inflammation reduction.

Finally, it is possible to speculate on novel potential therapeutic strategies, such as molecules able to inhibit NOX2 activation, that can be performed before surgery, as a sort of preconditioning, or even during the preparation of the conduits, to be used for the bypass. In this context, it has indeed been shown that modulation of NOX2-mediated oxidative stress improves endothelial function in CVD patients [36,46].

### 4.2. Limitations

The number of patients used in the study was small, and with a higher number the evidence would probably be stronger.

Second, we did not analyze patients with a coronary CT scan. This would have enabled better characterization of plaque and provided information on the possible qualitative association between plaque type and blood cytokines. However, although less precise, this study, which does not consider plaque differences, shows very strong results and may open up opportunities for that type of research.

Lastly, while this study confirms the hypothesized difference in oxidative stress/inflammation burden between coronary and internal thoracic arteries, the findings cannot be conclusive, as we did not directly measure blood “quality” after a coronary bypass was performed either at the level of the artery (due to technical reasons) or at the level of the coronary sinus (although feasible on CPB, we preferred to choose OP-CABG candidate patients to avoid the impact of CPB on oxidative stress/inflammation).

In conclusion, the novelty of this study lies in the discovery of higher NOX2-mediated oxidative stress and inflammation burden in blood drawn from a diseased coronary vessel, the LAD, vs. a healthy vessel, the LITA.

## 5. Conclusions

This study highlights the different burden of oxidative stress and inflammation markers between a LAD and a healthy LITA, on the same individual, showing local, organ-specific biological behaviour of coronary atherosclerosis. This could strengthen the base for the active biological targeting of coronary artery atherosclerosis.

Additional research is required to confirm the biological role of revascularization surgery, analysing coronary venous samples or myocardial tissue (muscle, fat) to confirm the beneficial subcellular effect. Further research on atherosclerosis disease peripheral biomarkers could lead towards personalized therapeutic targets and therapies able to positively modulate oxidative stress and inflammation.

## Figures and Tables

**Table 1:** Baseline patients’ characteristics.

Patients Characteristics	
N° of patients	20
Age, y	65 ± 10
Range	46–83
Male sex	18 (90)
BSA, m^2^	1.90 ± 0.16
BMI, kg/m^2^	26.1 ± 2.9
Familiar history of CAD	6 (30)
Smoking, n (%)	
Never	4 (20)
Former	10 (50)
Active	6 (30)
Hypertension	17 (85)
Diabetes mellitus, n (%)	
Type 1	2 (10)
Type 2	6 (30)
Diabetes therapy, n (%)	
Oral	2 (25)
Insulin	2 (25)
Oral + insulin	4 (50)
Dyslipidaemia	15 (75)
COPD	3 (15)
Paroxysmal AF	1 (5)
History of MI, n (%)	
NSTEMI	4 (20)
STEMI	2 (10)
Previous PTCA	5 (25)
Previous CVA	1 (5)
CAD presentation, n (%)	
STEMI	0
NSTEMI	2 (10)
Stable angina	14 (70)
Asymptomatic	4 (20)

Data are shown as mean ± standard deviation or frequencies (%). BSA: body surface area; BMI: body mass index; CAD: coronary artery disease; COPD: chronic obstructive pulmonary disease; AF: atrial fibrillation; MI: myocardial infarction; NSTEMI: non-ST-elevation myocardial infarction; STEMI: ST-elevation myocardial infarction; PTCA: percutaneous transcatheter coronary angioplasty; CVA: cerebrovascular accident.

**Table 2:** Preoperative echocardiographic and coronary angiography characteristics.

**Echocardiographic Characteristics**	
LVEF, %	56 ± 7
LVEDD, mm	46 ± 5
LVESD, mm	32 ± 5
IVS, mm	12 ± 2
PW, mm	10 ± 2
TAPSE, mm	26 ± 4
sPAP, mmHg	28 ± 9
**Coronary Angiography Characteristics**	
N° of diseased vessels	3.6 ± 1.0
Syntax Score	24 ± 5
LAD stenosis, %	85 ± 11

LVEF: left ventricle ejection fraction; LVEDD: left ventricle end-diastolic diameter; LEVSD: left ventricle end-systolic diameter; IVS: inter-ventricular septum; PW: posterior wall; TAPSE: tricuspid annulus plane systolic excursion; sPAP: systolic pulmonary artery pressure; LAD: left anterior descending.

## Data Availability

Anonymized metadata and raw data are available and can be accessed through a direct request to the corresponding author.

## Supplementary Materials

The following supporting information can be downloaded at: https://www.mdpi.com/article/10.3390/antiox13101180/s1, Supplemental Material S1: correlation and regression analyses; Table S1: patients’ laboratory data at admission; Table S2: patient therapy at admission.
