# Supplementary material for: Different Oxidative Stress and Inflammation Patterns of Diseased Left Anterior Descending Coronary Artery versus Internal Thoracic Artery
Source: Antioxidants (Basel). 2024 Sep 28;13(10):1180. doi: 10.3390/antiox13101180 (PMC11505158; doi:10.3390/antiox13101180)
Supplement: Supplementary file 1 [file antioxidants-13-01180-s001.zip › Supp Material S1.0.pdf]

Correlation and regression analysis

A correlation matrix was created to identify possible predictors or confounders for each laboratory outcome and univariate linear regression was performed. Any variable associated with the relative outcome with statistical significance (p) < 0.1 was considered for multivariate linear regression.

A correlation matrix was created to identify possible predictors or confounders for each laboratory outcome (Supplemental Table 3) and univariate linear regression was performed. Any variable associated with the relative outcome with statistical significance (p) < 0.1 was considered for multivariate linear regression. We found that the only independent predictive associations were: betablocker assumption for sNOX2-dp (LAD) levels (Beta Coef: -0.78; p: 0.005); NO (LITA) for NO (LAD) levels (Beta Coef: 0.646; p: 0.001) and viceversa (Beta Coef: 0.704; p: 0.001); Interleukin 1b (LAD) for Interleukin1b (LITA) levels (Beta Coef: 0.690; p: 0.045); female sex (Beta Coef: -0.637; p: <0.001) and Acetylsalicylic Acid assumption (Beta Coef: 0.566; p: 0.001) for Interleukin 10 (LITA).(Supplemental Table 4) The vast majority of preoperative variables were not related to laboratory findings. The association between NO and sNOX2-dp, like the association between IL1-β levels in the different vessels, are expected. Other associations (beta-blockers, acetylsalicylic acid or female sex) should be considered with caution due to the small number of patients.

Predictors	Statistics	Variables					
		LIMA_sNOx2-dp (pg/ml)	LAD_sNOx2-dp (pg/ml)	LIMA_H2O2 (µM)	LAD_H2O2 (µM)	LIMA_NO (µM)	LAD_NO (µM)
female sex	Pearson	-0,09	0,036	-0,248	0,166	0,02	-0,045
	P	0,706	0,879	0,292	0,485	0,935	0,85
height	Pearson	-0,182	-0,123	0,24	0,259	0,005	0,157
	P	0,47	0,627	0,338	0,3	0,985	0,535
weight	Pearson	-0,159	-0,38	0,059	-0,16	-0,142	-0,106
	P	0,529	0,12	0,815	0,526	0,575	0,676
BSA	Pearson	-0,018	-0,293	0,155	-0,196	-0,045	-0,007
	P	0,947	0,271	0,566	0,468	0,869	0,979
BMI	Pearson	-0,039	-0,384	-0,113	-0,452	-0,17	-0,271
	P	0,878	0,116	0,656	**0,06**	0,501	0,276
Family history	Pearson	-0,008	0,028	0,082	-0,195	0,021	-0,127
	P	0,974	0,905	0,731	0,411	0,93	0,593
smoke	Pearson	0,016	0,228	-0,079	-0,279	0,279	0,106
	P	0,947	0,335	0,741	0,234	0,234	0,657
hypertension	Pearson	0,141	-0,17	-0,311	-,491*	-0,328	-0,344
	P	0,553	0,474	0,182	**0,028**	0,159	0,138
diabetes	Pearson	0,196	0,105	-0,086	-0,206	0	0,048
	P	0,409	0,66	0,717	0,384	0,999	0,842
dyslipidemia	Pearson	0,243	-0,146	-0,443	-,470*	0,03	-0,006
	P	0,301	0,539	**0,051**	**0,037**	0,9	0,981
COPD	Pearson	-0,356	-0,435	0,017	-0,132	0,171	-0,007
	P	0,124	**0,055**	0,945	0,578	0,471	0,978
Obesity	Pearson	-0,046	-0,032	0,141	0,268	-0,08	-0,317
	P	0,846	0,894	0,553	0,253	0,739	0,173
Paroxysmal AF	Pearson	-0,385	-0,239	-0,172	0,17	-0,084	-0,312
	P	**0,094**	0,311	0,468	0,473	0,725	0,181
Prior AMI	Pearson	-0,278	-0,021	-0,037	-0,173	0,111	0,083
	P	0,235	0,929	0,878	0,467	0,641	0,728
Prior PTCA	Pearson	-0,101	0,098	0,048	-0,268	0,16	0,245
	P	0,672	0,682	0,841	0,253	0,501	0,298
presentation - NSTEMI	Pearson	-0,335	-,469*	-0,175	-0,067	-0,191	-0,418

Predictors	Statistics	Variables					
		LIMA_sNOx2-dp (pg/ml)	LAD_sNOx2-dp (pg/ml)	LIMA_H2O2 (µM)	LAD_H2O2 (µM)	LIMA_NO (µM)	LAD_NO (µM)
	P	0,149	**0,037**	0,461	0,78	0,419	**0,067**
presentation - Unstable Angina	Pearson	0,165	0,271	-0,142	-0,014	0,122	0,138
	P	0,487	0,248	0,55	0,954	0,61	0,562
presentation - asymptomatic	Pearson	0,062	0,041	0,294	0,066	0,004	0,155
	P	0,795	0,862	0,208	0,782	0,986	0,513
RBC	Pearson	-0,14	0,285	0,18	0,264	-0,142	0,009
	P	0,566	0,236	0,46	0,274	0,563	0,97
Hb	Pearson	-0,275	0,219	0,022	0,146	-0,04	0,128
	P	0,254	0,369	0,929	0,55	0,872	0,602
Htc	Pearson	-0,227	0,331	0,11	0,176	0,034	0,228
	P	0,351	0,166	0,655	0,471	0,889	0,347
WBC	Pearson	0,198	0,107	-0,172	0,296	-0,102	-0,118
	P	0,415	0,662	0,482	0,218	0,677	0,631
Neutrofils %	Pearson	0,242	-0,244	-0,033	-0,011	-0,29	-0,217
	P	0,319	0,315	0,893	0,964	0,228	0,373
Lymphocytes %	Pearson	-0,169	0,355	0,109	0,078	0,24	0,144
	P	0,489	0,136	0,657	0,75	0,322	0,557
Eosinofils %	Pearson	0,041	-0,137	-,514*	-0,181	-0,09	-0,11
	P	0,869	0,576	**0,025**	0,458	0,715	0,655
Basofils %	Pearson	0,012	0,357	0,241	0,441	0,331	,534*
	P	0,963	0,134	0,321	**0,059**	0,166	**0,018**
Monocytes %	Pearson	-0,399	-0,28	-0,078	-0,219	0,309	0,305
	P	**0,091**	0,246	0,75	0,369	0,198	0,205
PLT	Pearson	0,027	0,351	0,104	,687**	-0,159	-0,1
	P	0,913	0,14	0,672	**0,001**	0,516	0,683
creatinine	Pearson	0,123	-0,223	0,181	-0,164	0,229	0,138
	P	0,615	0,359	0,46	0,502	0,345	0,575
Glycemia	Pearson	0,238	-0,188	-0,322	-0,263	-0,116	-0,171
	P	0,327	0,441	0,179	0,277	0,636	0,484
HB Gly Fraction	Pearson	0,33	0,165	-0,205	0,002	-0,064	-0,084
	P	0,196	0,526	0,43	0,994	0,807	0,748

Predictors	Statistics	Variables					
		LIMA_sNOx2-dp (pg/ml)	LAD_sNOx2-dp (pg/ml)	LIMA_H2O2 (µM)	LAD_H2O2 (µM)	LIMA_NO (µM)	LAD_NO (µM)
Hb Glyc Tot	Pearson	0,318	0,154	-0,214	0,009	-0,051	-0,079
	P	0,213	0,556	0,41	0,974	0,845	0,763
Cholesterol tot	Pearson	-0,093	-0,062	0,271	0,171	-0,05	-0,047
	P	0,713	0,806	0,276	0,497	0,844	0,854
Cholesterol LDL	Pearson	-0,192	-0,057	0,246	0,087	-0,124	-0,159
	P	0,446	0,821	0,325	0,732	0,625	0,528
Cholesterol HDL	Pearson	0,43	0,398	0,226	0,255	0,18	0,445
	P	**0,075**	0,102	0,368	0,307	0,474	**0,064**
Triglyceridis	Pearson	-0,129	-0,079	0,3	0,383	0,258	0,355
	P	0,611	0,757	0,226	0,117	0,301	0,148
AST (GOT)	Pearson	-0,201	0,214	0,1	0,169	-0,001	-0,251
	P	0,41	0,38	0,684	0,488	0,997	0,3
BUN	Pearson	0,101	-0,199	0,229	0,055	0,377	0,172
	P	0,682	0,414	0,345	0,822	0,112	0,481
CRP	Pearson	-0,433	-0,294	-0,146	0,216	-0,028	-0,41
	P	**0,064**	0,222	0,551	0,374	0,91	**0,081**
ESV	Pearson	-0,327	-0,467	0,019	-0,062	-0,041	-0,446
	P	0,253	**0,092**	0,949	0,833	0,889	0,11
CK tot	Pearson	0,107	-0,21	0,168	-0,419	0,308	0,2
	P	0,664	0,388	0,492	**0,074**	0,2	0,413
CK MB	Pearson	-0,039	0,006	0,308	-0,322	0,221	0,161
	P	0,874	0,981	0,2	0,179	0,364	0,511
Troponine T	Pearson	-0,17	-,492*	-0,123	-0,303	-0,124	-0,414
	P	0,486	**0,032**	0,615	0,207	0,614	**0,078**
Diseased coronary vessels (n°)	Pearson	-0,213	0,163	-0,176	0,078	-0,362	-0,298
	P	0,368	0,492	0,457	0,742	0,117	0,202
% LAD stenosis	Pearson	-0,415	-0,108	0,061	0,443	-0,047	-0,122
	P	**0,069**	0,65	0,798	**0,05**	0,843	0,609
Syntax score	Pearson	0,173	0,31	0,199	-0,192	-0,212	-0,14
	P	0,573	0,303	0,514	0,529	0,487	0,648
PPI	Pearson	0,205	-0,043	-0,229	0,14	,517*	0,235

Predictors	Statistics	Variables					
		LIMA_sNOx2-dp (pg/ml)	LAD_sNOx2-dp (pg/ml)	LIMA_H2O2 (µM)	LAD_H2O2 (µM)	LIMA_NO (µM)	LAD_NO (µM)
	P	0,385	0,856	0,331	0,555	**0,02**	0,319
ASA	Pearson	-0,055	-0,086	-0,394	-0,068	0,167	-0,162
	P	0,817	0,72	**0,086**	0,777	0,483	0,496
PLAVIX	Pearson	-0,215	-0,242	-0,42	-0,299	0,053	0,069
	P	0,362	0,303	**0,065**	0,201	0,823	0,771
BB	Pearson	0,054	-,563**	-0,232	0,083	-0,225	-0,219
	P	0,822	**0,01**	0,325	0,727	0,34	0,354
ACEI	Pearson	0,436	-0,058	0,165	-0,113	-0,21	0,067
	P	**0,055**	0,808	0,488	0,635	0,375	0,778
ARB	Pearson	-0,245	-0,186	-0,166	-0,068	0,004	-0,192
	P	0,298	0,432	0,485	0,775	0,985	0,416
CCB	Pearson	-0,032	0,275	-0,03	-0,073	0,161	-0,162
	P	0,893	0,241	0,899	0,761	0,499	0,495
Diuretics	Pearson	0,005	-0,24	0,047	-0,137	0,266	0,281
	P	0,983	0,308	0,843	0,564	0,257	0,23
Statins	Pearson	0,142	-0,082	-0,402	-0,343	0,149	0,064
	P	0,55	0,732	**0,079**	0,139	0,53	0,79
Nitro deriv	Pearson	-0,389	-0,128	-0,138	-0,029	-0,285	-,550*
	P	**0,09**	0,59	0,562	0,903	0,223	**0,012**
Ejection Fraction	Pearson	0	-0,028	-0,193	-0,052	-0,254	-0,419
	P	0,998	0,908	0,414	0,826	0,281	**0,066**
LVEDD	Pearson	0,204	-0,03	0,326	-0,061	0,385	0,354
	P	0,416	0,905	0,187	0,809	0,115	0,15
LVESD	Pearson	0,096	0,118	,525*	0,212	0,326	0,452
	P	0,724	0,663	**0,037**	0,431	0,219	**0,079**
IVS	Pearson	-,488*	-0,071	0,143	-0,234	-,547*	-0,374
	P	**0,04**	0,779	0,572	0,349	**0,019**	0,126
PW	Pearson	-0,056	-0,394	0,013	-0,302	-,555*	-0,238
	P	0,826	0,105	0,958	0,224	**0,017**	0,341
TAPSE	Pearson	-0,027	-0,125	0,025	0,145	-0,248	-0,441
	P	0,934	0,698	0,938	0,652	0,437	0,151

Predictors	Statistics	Variables					
		LIMA_sNOx2-dp (pg/ml)	LAD_sNOx2-dp (pg/ml)	LIMA_H2O2 (µM)	LAD_H2O2 (µM)	LIMA_NO (µM)	LAD_NO (µM)
PAPS	Pearson	0,209	-0,2	-0,112	-0,161	-0,202	-0,04
	P	0,404	0,425	0,659	0,524	0,422	0,876
LIMA_sNOx2-dp (pg/ml)	Pearson	1	-0,105	-0,204	-0,287	0,141	0,247
	P		0,659	0,387	0,22	0,554	0,294
LAD_sNOx2-dp (pg/ml)	Pearson	-0,105	1	0,381	0,382	0,073	0,193
	P	0,659		**0,098**	**0,097**	0,76	0,416
LIMA_H2O2 (µM)	Pearson	-0,204	0,381	1	0,36	0,077	0,222
	P	0,387	**0,098**		0,119	0,746	0,346
LAD_H2O2 (µM)	Pearson	-0,287	0,382	0,36	1	-0,043	0
	P	0,22	**0,097**	0,119		0,858	0,998
LIMA_NO (µM)	Pearson	0,141	0,073	0,077	-0,043	1	,734**
	P	0,554	0,76	0,746	0,858		**0**
LAD_NO (µM)	Pearson	0,247	0,193	0,222	0	,734**	1
	P	0,294	0,416	0,346	0,998	**0**	
LIMA_TNF-alpha	Pearson	-0,266	0,129	-0,035	0,191	-0,151	-0,229
	P	0,258	0,587	0,884	0,42	0,525	0,332
LAD_TNF-alpha	Pearson	-0,006	0,048	-0,429	-0,13	0,085	-0,126
	P	0,978	0,839	**0,059**	0,586	0,722	0,596
LIMA_IL-6 (pg/ml)	Pearson	0,206	0,058	0,193	-0,221	-0,209	-0,096
	P	0,384	0,808	0,415	0,348	0,377	0,686
LAD_IL-6 (pg/ml)	Pearson	0,069	0,12	0,085	0,047	0,133	-0,246
	P	0,774	0,614	0,72	0,845	0,576	0,296
LIMA_IL-1b (pg/ml)	Pearson	-0,396	-0,095	,492*	0,054	-0,032	0,001
	P	**0,084**	0,69	**0,028**	0,822	0,894	0,996
LAD_IL-1b (pg/ml)	Pearson	0,011	-0,281	0,239	-0,128	0,049	0,099
	P	0,963	0,23	0,309	0,591	0,838	0,678
LIMA_IL-10 (pg/ml)	Pearson	-0,277	-0,123	0,009	-0,064	-0,021	-0,085
	P	0,237	0,604	0,971	0,789	0,929	0,723
LAD_IL-10 (pg/ml)	Pearson	-,655**	0,13	0,087	0,079	-0,127	-0,193
	P	**0,002**	0,586	0,715	0,739	0,593	0,415

Predictors	Statistics	Variables							
		LIMA_TNF-alpha	LAD_TNF-alpha	LIMA_IL-6 (pg/ml)	LAD_IL-6 (pg/ml)	LIMA_IL-1b (pg/ml)	LAD_IL-1b (pg/ml)	LIMA_IL-10 (pg/ml)	LAD_IL-10 (pg/ml)
female sex	Pearson	0,043	0,286	-0,164	-0,221	0,025	-0,067	-0,38	0,335
	P	0,856	0,221	0,491	0,349	0,916	0,78	**0,098**	0,149
height	Pearson	0,248	-0,396	-0,435	0,135	0,063	-0,03	-0,202	-0,1
	P	0,32	0,103	**0,071**	0,594	0,803	0,907	0,421	0,694
weight	Pearson	0,281	-0,261	-0,117	-0,048	-0,214	-0,222	0,174	-0,141
	P	0,259	0,295	0,643	0,851	0,393	0,376	0,489	0,578
BSA	Pearson	0,318	-0,413	-0,245	0,119	-0,119	-0,057	-0,006	-0,181
	P	0,23	0,112	0,361	0,662	0,661	0,833	0,982	0,503
BMI	Pearson	0,153	0,022	0,264	-0,19	-0,321	-0,218	0,397	-0,104
	P	0,545	0,93	0,289	0,449	0,193	0,384	0,103	0,68
Family history	Pearson	0,025	0,095	-0,035	0,139	-0,056	0,272	,462*	0,248
	P	0,918	0,691	0,883	0,56	0,816	0,246	**0,04**	0,291
smoke	Pearson	-0,304	-0,013	-0,197	0,026	-0,005	-0,354	-0,057	0,061
	P	0,193	0,957	0,405	0,913	0,983	0,125	0,81	0,797
hypertension	Pearson	0,037	-0,164	0,061	0,064	-0,228	-0,304	0,068	0,253
	P	0,878	0,49	0,799	0,789	0,333	0,193	0,775	0,283
diabetes	Pearson	-0,086	-0,324	0,418	0,149	-0,251	-0,332	0,031	-0,201
	P	0,717	0,163	**0,067**	0,53	0,287	0,153	0,895	0,397
dyslipidemia	Pearson	-0,107	-0,2	0,066	-0,207	-0,419	-0,313	0,013	-0,008
	P	0,653	0,399	0,783	0,381	**0,066**	0,178	0,955	0,974
COPD	Pearson	-0,165	-0,317	-0,106	-0,016	0,348	-0,029	-0,05	0,24
	P	0,487	0,173	0,655	0,945	0,132	0,904	0,835	0,309
Obesity	Pearson	0,073	0,133	-0,28	,503*	-0,001	-0,181	0,158	0,129
	P	0,761	0,576	0,232	**0,024**	0,998	0,446	0,506	0,589
Paroxysmal AF	Pearson	0,08	-0,048	-0,25	-0,04	0,247	0,156	-0,122	,476*
	P	0,736	0,842	0,287	0,869	0,295	0,51	0,607	**0,034**
Prior AMI	Pearson	-0,112	-0,296	-0,377	-0,034	0,203	0,344	0,355	0,438
	P	0,638	0,205	0,102	0,886	0,392	0,138	0,125	**0,053**
Prior PTCA	Pearson	-0,159	-0,29	-0,273	-0,016	0,09	0,285	0,437	0,224
	P	0,502	0,216	0,245	0,945	0,705	0,223	**0,054**	0,342
presentation - NSTEMI	Pearson	0,172	0,154	0,013	-0,313	0,156	0,21	0,053	0,422

Predictors	Statistics	Variables							
		LIMA_TNF-alpha	LAD_TNF-alpha	LIMA_IL-6 (pg/ml)	LAD_IL-6 (pg/ml)	LIMA_IL-1b (pg/ml)	LAD_IL-1b (pg/ml)	LIMA_IL-10 (pg/ml)	LAD_IL-10 (pg/ml)
	P	0,468	0,516	0,958	0,18	0,512	0,373	0,825	**0,064**
presentation - Unstable Angina	Pearson	-0,055	0,123	-0,339	0,182	-,480*	-0,26	0,139	-0,288
	P	0,819	0,607	0,143	0,442	**0,032**	0,268	0,558	0,218
presentation - asymptomatic	Pearson	-0,066	-0,256	0,379	0,026	0,433	0,14	-0,199	0,014
	P	0,781	0,276	**0,099**	0,914	**0,056**	0,555	0,4	0,955
RBC	Pearson	0,087	-0,126	-0,013	-0,155	-0,251	-0,181	0,285	-0,096
	P	0,722	0,607	0,958	0,525	0,299	0,459	0,236	0,696
Hb	Pearson	0,103	-0,08	0,093	-,536*	-0,061	-0,004	0,092	-0,038
	P	0,674	0,745	0,706	**0,018**	0,804	0,987	0,708	0,877
Htc	Pearson	0,022	-0,107	0,046	-,466*	-0,061	-0,046	0,112	-0,057
	P	0,929	0,663	0,853	**0,044**	0,803	0,851	0,648	0,818
WBC	Pearson	0,104	0,134	-0,016	0,051	-0,391	-0,233	0,264	-0,26
	P	0,673	0,584	0,949	0,835	**0,098**	0,337	0,275	0,282
Neutrofils %	Pearson	-0,01	0,184	0,216	0,265	-0,105	-0,15	0,296	0,019
	P	0,967	0,45	0,375	0,273	0,669	0,541	0,219	0,94
Lymphocytes %	Pearson	0,027	-0,117	-0,14	-0,162	0,068	0,106	-0,382	-0,085
	P	0,912	0,632	0,568	0,509	0,782	0,667	0,107	0,728
Eosinofils %	Pearson	0,262	0,039	-0,102	-0,157	-0,376	-0,204	-0,287	-0,131
	P	0,279	0,874	0,679	0,52	0,112	0,401	0,234	0,593
Basofils %	Pearson	-0,208	0,155	-0,366	-0,242	0,165	0,096	-0,198	-0,108
	P	0,392	0,526	0,123	0,318	0,501	0,695	0,415	0,661
Monocytes %	Pearson	-0,146	-0,318	-0,255	-0,368	0,348	0,327	0,277	0,293
	P	0,551	0,185	0,292	0,121	0,144	0,171	0,251	0,224
PLT	Pearson	0,058	0,114	0,088	0,3	-0,255	-0,245	0,134	-0,126
	P	0,813	0,644	0,721	0,212	0,292	0,313	0,583	0,606
creatinine	Pearson	0,183	-0,425	-0,112	0,134	0,088	0,053	0,025	-0,233
	P	0,455	**0,07**	0,647	0,584	0,722	0,83	0,919	0,337
Glycemia	Pearson	0,108	-0,127	-0,047	0,055	-0,32	-0,276	-0,134	-0,397
	P	0,659	0,605	0,85	0,822	0,182	0,252	0,585	**0,092**
HB Gly Fraction	Pearson	0,015	-0,352	0,09	0,314	-0,416	-0,362	-0,195	-0,427
	P	0,954	0,166	0,731	0,219	**0,097**	0,153	0,454	**0,087**

Predictors	Statistics	Variables							
		LIMA_TNF-alpha	LAD_TNF-alpha	LIMA_IL-6 (pg/ml)	LAD_IL-6 (pg/ml)	LIMA_IL-1b (pg/ml)	LAD_IL-1b (pg/ml)	LIMA_IL-10 (pg/ml)	LAD_IL-10 (pg/ml)
Hb Glyc Tot	Pearson	0,002	-0,354	0,083	0,332	-0,41	-0,369	-0,176	-0,433
	P	0,994	0,164	0,753	0,193	0,102	0,145	0,499	**0,082**
Cholesterol tot	Pearson	0,111	-0,065	-0,192	-,542*	0,279	0,274	-0,017	0,176
	P	0,661	0,797	0,445	**0,02**	0,263	0,271	0,946	0,485
Cholesterol LDL	Pearson	0,168	0,002	-0,132	-,525*	0,293	0,26	0,05	0,276
	P	0,505	0,994	0,6	**0,025**	0,238	0,297	0,843	0,268
Cholesterol HDL	Pearson	-0,25	-0,175	0,031	-0,055	-0,084	0,018	-0,175	-0,162
	P	0,317	0,488	0,904	0,83	0,74	0,943	0,487	0,521
Triglyceridis	Pearson	0,246	-0,444	-,515*	-0,378	0,19	0,068	-0,149	-0,079
	P	0,325	**0,065**	**0,029**	0,122	0,451	0,789	0,556	0,756
AST (GOT)	Pearson	-0,091	-0,067	0,207	0,229	-0,076	-0,318	-0,02	0,262
	P	0,71	0,784	0,396	0,346	0,757	0,184	0,934	0,279
BUN	Pearson	0,048	-0,272	-0,268	0,389	0,047	-0,085	-0,216	-0,096
	P	0,846	0,261	0,268	0,1	0,847	0,729	0,374	0,695
CRP	Pearson	0,194	0,175	-0,262	0,132	0,23	0,119	-0,021	,518*
	P	0,427	0,473	0,278	0,591	0,343	0,628	0,933	**0,023**
ESV	Pearson	0,145	0,069	0,275	-0,106	0,419	0,134	-0,106	0,401
	P	0,621	0,814	0,341	0,719	0,136	0,648	0,719	0,155
CK tot	Pearson	-0,031	-0,184	,605**	0,142	-0,084	-0,124	0,055	-0,067
	P	0,901	0,45	**0,006**	0,563	0,731	0,613	0,822	0,785
CK MB	Pearson	0,107	-0,084	0,381	0,183	-0,078	-0,272	0,015	0,123
	P	0,663	0,733	0,107	0,453	0,752	0,261	0,953	0,616
Troponine T	Pearson	0,239	0,176	0,257	-0,263	0,05	0,103	-0,026	0,223
	P	0,324	0,472	0,287	0,276	0,839	0,674	0,917	0,359
Diseased coronary vessels (n°)	Pearson	0,361	-0,036	-0,059	0,111	-0,096	-0,094	0,065	,451*
	P	0,118	0,881	0,803	0,641	0,688	0,695	0,784	**0,046**
% LAD stenosis	Pearson	0,414	0,216	0,034	-0,011	-0,033	-0,108	0,085	0,142
	P	**0,069**	0,359	0,888	0,965	0,891	0,652	0,721	0,549
Syntax score	Pearson	0,069	-0,133	,570*	0,346	0,059	0,183	-0,126	0,056
	P	0,822	0,664	**0,042**	0,247	0,849	0,551	0,683	0,855
PPI	Pearson	-0,2	0,004	-0,249	-0,014	-,458*	-0,305	0,073	-0,3

Predictors	Statistics	Variables							
		LIMA_TNF-alpha	LAD_TNF-alpha	LIMA_IL-6 (pg/ml)	LAD_IL-6 (pg/ml)	LIMA_IL-1b (pg/ml)	LAD_IL-1b (pg/ml)	LIMA_IL-10 (pg/ml)	LAD_IL-10 (pg/ml)
	P	0,397	0,985	0,289	0,954	**0,042**	0,191	0,76	0,198
ASA	Pearson	-0,097	0,289	-0,045	0,068	-0,386	-0,355	,537*	0,086
	P	0,685	0,216	0,852	0,777	**0,093**	0,125	**0,015**	0,718
PLAVIX	Pearson	-0,047	0,175	-0,307	-0,148	-0,059	-0,144	0,158	-0,11
	P	0,845	0,461	0,188	0,532	0,806	0,546	0,505	0,644
BB	Pearson	0,021	-0,113	-0,383	-0,003	0,175	0,341	0,205	-0,114
	P	0,929	0,635	**0,096**	0,99	0,461	0,141	0,386	0,632
ACEI	Pearson	-,582**	-0,192	0,126	-0,121	0,298	,463*	0,055	-0,313
	P	**0,007**	0,419	0,596	0,611	0,202	**0,04**	0,818	0,178
ARB	Pearson	,466*	-0,002	0,006	0,086	-0,28	-,491*	0,129	0,35
	P	**0,038**	0,994	0,98	0,718	0,232	**0,028**	0,589	0,13
CCB	Pearson	0,269	-0,055	0,032	0,411	-,458*	-,485*	0,159	-0,057
	P	0,251	0,818	0,894	**0,072**	**0,042**	**0,03**	0,502	0,811
Diuretics	Pearson	0,075	-0,385	-0,07	-0,133	0,071	-0,107	-0,188	-0,252
	P	0,752	**0,094**	0,77	0,576	0,765	0,655	0,427	0,283
Statins	Pearson	-0,341	0,08	0,159	-0,231	-0,369	-0,211	0,15	-0,205
	P	0,141	0,739	0,504	0,328	0,109	0,372	0,529	0,387
Nitro deriv	Pearson	0,181	0,153	0,003	-0,19	0,047	0,092	-0,048	0,372
	P	0,446	0,519	0,989	0,422	0,845	0,698	0,84	0,107
Ejection Fraction	Pearson	-0,16	0,215	0,23	0,098	-0,307	-,450*	0,181	0,111
	P	0,5	0,363	0,33	0,68	0,187	**0,046**	0,444	0,64
LVEDD	Pearson	0,334	-0,249	-0,161	0,152	0,118	0,357	-0,256	-0,184
	P	0,176	0,318	0,525	0,546	0,64	0,145	0,305	0,465
LVESD	Pearson	0,342	-0,368	-0,19	0,129	0,219	0,358	-0,318	-0,084
	P	0,195	0,161	0,48	0,634	0,415	0,173	0,23	0,756
IVS	Pearson	0,379	0,039	0,042	-0,167	0,284	0,095	0,04	,502*
	P	0,121	0,878	0,867	0,507	0,254	0,707	0,874	**0,034**
PW	Pearson	0,252	-0,004	0,032	-0,265	0,25	0,296	-0,147	0,044
	P	0,313	0,989	0,901	0,288	0,318	0,233	0,56	0,863
TAPSE	Pearson	0,074	0,383	-0,401	0,307	-0,035	-0,217	0,059	0,158
	P	0,818	0,22	0,197	0,332	0,915	0,498	0,856	0,624

Predictors	Statistics	Variables							
		LIMA_TNF-alpha	LAD_TNF-alpha	LIMA_IL-6 (pg/ml)	LAD_IL-6 (pg/ml)	LIMA_IL-1b (pg/ml)	LAD_IL-1b (pg/ml)	LIMA_IL-10 (pg/ml)	LAD_IL-10 (pg/ml)
PAPS	Pearson	-0,15	-0,066	-0,059	0,164	-0,119	-0,132	0,229	-0,192
	P	0,552	0,795	0,817	0,515	0,639	0,602	0,362	0,446
LIMA_sNOx2-dp (pg/ml)	Pearson	-0,266	-0,006	0,206	0,069	-0,396	0,011	-0,277	-,655**
	P	0,258	0,978	0,384	0,774	**0,084**	0,963	0,237	**0,002**
LAD_sNOx2-dp (pg/ml)	Pearson	0,129	0,048	0,058	0,12	-0,095	-0,281	-0,123	0,13
	P	0,587	0,839	0,808	0,614	0,69	0,23	0,604	0,586
LIMA_H2O2 (µM)	Pearson	-0,035	-0,429	0,193	0,085	,492*	0,239	0,009	0,087
	P	0,884	**0,059**	0,415	0,72	**0,028**	0,309	0,971	0,715
LAD_H2O2 (µM)	Pearson	0,191	-0,13	-0,221	0,047	0,054	-0,128	-0,064	0,079
	P	0,42	0,586	0,348	0,845	0,822	0,591	0,789	0,739
LIMA_NO (µM)	Pearson	-0,151	0,085	-0,209	0,133	-0,032	0,049	-0,021	-0,127
	P	0,525	0,722	0,377	0,576	0,894	0,838	0,929	0,593
LAD_NO (µM)	Pearson	-0,229	-0,126	-0,096	-0,246	0,001	0,099	-0,085	-0,193
	P	0,332	0,596	0,686	0,296	0,996	0,678	0,723	0,415
LIMA_TNF-alpha	Pearson	1	-0,014	-0,074	0,206	-0,29	-0,176	-0,023	0,365
	P		0,952	0,755	0,384	0,215	0,457	0,923	0,114
LAD_TNF-alpha	Pearson	-0,014	1	0,122	-0,058	-0,269	-0,132	-0,088	-0,032
	P	0,952		0,609	0,808	0,251	0,58	0,713	0,892
LIMA_IL-6 (pg/ml)	Pearson	-0,074	0,122	1	-0,042	-0,106	-0,047	-0,113	-0,251
	P	0,755	0,609		0,86	0,656	0,843	0,635	0,286
LAD_IL-6 (pg/ml)	Pearson	0,206	-0,058	-0,042	1	-0,012	-0,052	0,105	0,091
	P	0,384	0,808	0,86		0,959	0,827	0,66	0,701
LIMA_IL-1b (pg/ml)	Pearson	-0,29	-0,269	-0,106	-0,012	1	,661**	0,16	0,403
	P	0,215	0,251	0,656	0,959		**0,002**	0,499	**0,078**
LAD_IL-1b (pg/ml)	Pearson	-0,176	-0,132	-0,047	-0,052	,661**	1	0,167	0,117
	P	0,457	0,58	0,843	0,827	**0,002**		0,482	0,622
LIMA_IL-10 (pg/ml)	Pearson	-0,023	-0,088	-0,113	0,105	0,16	0,167	1	0,406
	P	0,923	0,713	0,635	0,66	0,499	0,482		**0,076**
LAD_IL-10 (pg/ml)	Pearson	0,365	-0,032	-0,251	0,091	0,403	0,117	0,406	1
	P	0,114	0,892	0,286	0,701	**0,078**	0,622	**0,076**	

	LIMA_sNOx2-dp	LAD_sNOx2-dp	LIMA_H2O2	LAD_H2O2	LIMA_NO	LAD_NO	LIMA_TNF-a	LAD_TNF-a	LIMA_IL-6	LAD_IL-6	LIMA_IL-1b	LAD_IL-1b	LIMA_IL-10	LAD_IL-10
**Multivariate analysis Predictors**	none	**BB (betablockers)**<br>Beta Coef: -0.78<br>p: 0.005	none	none	**LAD_NO**<br>Beta Coef: 0.646<br>p: 0.001	**LIMA_NO**<br>Beta Coef: 0.704<br>p: 0.001	none	none	none	none	**LAD_IL-1b**<br>Beta Coef: 0.690<br>p: 0.045	none	**Female Sex**<br>Beta Coef: -0.637<br>p: <0.001<br>**ASA (acetylsalicylic acid)**<br>Beta Coef: 0.566<br>p: 0.001	none
