# Supplementary material for: Different Oxidative Stress and Inflammation Patterns of Diseased Left Anterior Descending Coronary Artery versus Internal Thoracic Artery
Source: Antioxidants (Basel). 2024 Sep 28;13(10):1180. doi: 10.3390/antiox13101180 (PMC11505158; doi:10.3390/antiox13101180)
Supplement: Supplementary file 1 [file antioxidants-13-01180-s001.zip › Table S1.pdf]

Supplemental Table S1. Laboratory findings at admission.

**Blood analysis**	**Value**
RBC, *10^6/µl*	4.45 ± 0.51
Hb, *g/dl*	13.6 ± 1.8
Htc, %	40.8 ± 5.4
WBC, *10^3/µl*	8.42 ± 2.25
Neutrophils, %	64.4 ± 11.3
Lymphocytes, %	26.2 ± 9.8
Monocytes, %	7.2 ± 2.8
Eosinophils, %	1.4 ± 1.3
Basophils, %	0.5 ± 0.3
Platelets, *10^3/µl*	228 ± 57
Creatinine, *mg/dl*	1.01 ± 0.21
Urea, *mg/dl*	38.9 ± 12.6
Glycemia, *mg/dl*	105 ± 29
Hb glyc, %	5.9 ± 0.7
Hb glyc, *mmol/mol*	41.5 ± 8.4
Total Cholesterol, *mg/dl*	147 ± 47
LDL, *mg/dl*	86 ± 43
HDL, *mg/dl*	42 ± 10
Triglycerides, *mg/dl*	164 ± 126
GPT, *u/l*	22 ± 9
GOT, *u/l*	19 ± 5
ESR, *s*	22 ± 23
CRP, *mg/dl*	0.71 ± 1.14
CK, *u/l*	110 ± 81
CK-MB, *u/l*	3.0 ± 1.8
T-Troponin, *pg/ml*	26.9 ± 32.9

RBC: red blood cells; Hb: haemoglobin; Hct: haematocrit; WBC: white blood cells; Hb glyc: glycosylate haemoglobin; LDL: low-density lipoprotein; HDL: high-density lipoprotein; GPT: glutamate pyruvate transaminase; GOT: glutamic oxaloacetic transaminase; ESR: erythrocyte sedimentation rate; CRP: C-reactive protein; CK: creatinkinase; CK-MB: creatinkinase MB
