# Supplementary material for: Different Oxidative Stress and Inflammation Patterns of Diseased Left Anterior Descending Coronary Artery versus Internal Thoracic Artery
Source: Antioxidants (Basel). 2024 Sep 28;13(10):1180. doi: 10.3390/antiox13101180 (PMC11505158; doi:10.3390/antiox13101180)
Supplement: Supplementary file 1 [file antioxidants-13-01180-s001.zip › Table S2.pdf]

Supplementary Table S2. Patient therapy at admission.

Drug	Prevalence
PPI	19 (95)
ASA	17 (85)
Clopidogrel	3 (15)
B-blockers	16 (80)
ACE-I	7 (35)
ARB	10 (50)
CCB	9 (45)
Diuretics	4 (20)
Statins	17 (85)
Nitrates	3 (15)

PPI: proton pump inhibitor; ASA: acetylsalicylic acid; ACE-I: angiotensin converting enzyme inhibitor; ARB: angiotensin receptor blocker; CCB: calcium channel blocker
